# Superior fornix mass with retained soft contact lens

**Published:** 2020-12-31

**Authors:** Carl Benjamin Rebhun, Ann Q Tran, Irina Belinsky

**Affiliations:** Department of Ophthalmology, NYU Langone Health, New York, USA

**Keywords:** Contact lens, foreign body granuloma, oculoplastics

A 35-year-old female presented to the oculoplastics clinic for a 1-month history of left upper eyelid painful mass, ptosis, and superior conjunctivitis with discharge [Fig. 1a]. A palpable, pyogenic-like mass was present in the deep superior fornix. Computed tomography imaging demonstrated a 1.2 cm well-circumscribed lesion superficially in the upper eyelid [Fig. 1b]. Intraoperatively, double eversion of the eyelid and dissection into the mass revealed a large pocket granuloma with a retained soft contact lens [Fig. 1c].

## Discussion

Forniceal pyogenic granulomas involving contact lenses are usually a result of rigid gas permeable lenses, and generally cause chronic conjunctivitis and eye irritation.^[1]^ Less commonly, retained soft contact lenses have been reported to masquerade as a superior orbital mass.^[2]^ In these cases, double eversion of the eyelid generally reveals a visible contact lens in the deep superior fornix. In our case, double eversion revealed a deep forniceal defect with the contact lens surrounded by granulomatous tissue. Computed tomography imaging of masses with retained contact lens generally reveals a homogeneous cyst-like mass without evidence of a foreign body.^[3]^ Some authors hypothesized that after a contact lens is displaced into the upper fornix, the contact lens can become fixed against the upper border of the tarsus.^[4]^ With blinking, the contact lens erodes through the conjunctiva and can eventually be surrounded by granulation tissue.^[5]^

Regardless of a history of a lost contact lens, the presence of a soft, mobile, purulent mass in the deep fornix of a contact lens wearer should raise suspicion for a foreign body granuloma from a retained contact lens. High clinical suspicion is key to establishing this relatively rare diagnosis given that some cases may present without conjunctival irritation or injection.^[5]^ Surgical removal of the foreign body granuloma with a course of topical antibiotics can result in good clinical outcomes. Prevention of contact lens related complication through patient education cannot be overemphasized.

## Figures and Tables

**Figure 1:**
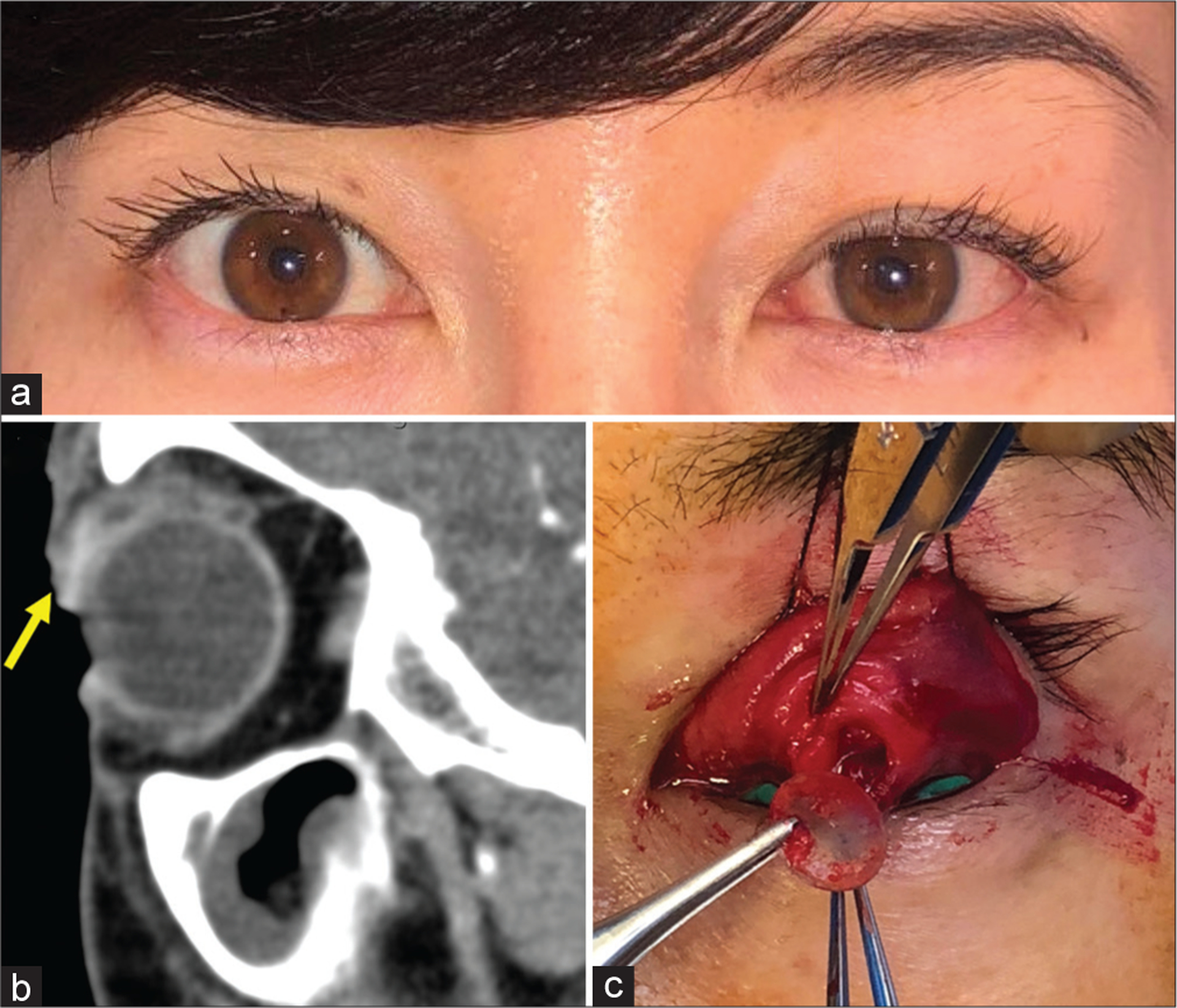
A case of a retained contact lens causing foreign body granuloma. (a) External photograph demonstrating left eyelid fullness, ptosis, and conjunctival injection. (b) Computed tomography sagittal cut demonstrating a well‑circumscribed, centrally low‑density cystic lesion (yellow arrow) in the anterosuperior margin of the left globe. (c) Intraoperative photograph demonstrating everted superior eyelid with a deep forniceal defect containing a foreign body granuloma and removal of soft contact lens
